# *Trichinella* spp. in wild boar (*Sus scrofa*) populations in Croatia during an eight-year study (2010–2017)

**DOI:** 10.1016/j.onehlt.2020.100172

**Published:** 2020-09-29

**Authors:** Davor Balić, Gianluca Marucci, Marija Agičić, Miroslav Benić, Zlatko Krovina, Tihana Miškić, Krunoslav Aladić, Mario Škrivanko

**Affiliations:** a Croatian Veterinary Institute, Veterinary Department Vinkovci, J. Kozarca 24, 32100 Vinkovci, Croatia; b Istituto Superiore di Sanità, Viale Regina Elena 299, 00161, Rome, Italy; c Croatian Veterinary Institute, Department for Bacteriology and Parasitology, Savska 143, 10000 Zagreb, Croatia; d Ministry of Agriculture, Planinska ulica 2a, 10000 Zagreb, Croatia

**Keywords:** Croatia, wild boar, *Trichinella* spp., Mixed infection, ESV region, Sequencing

## Abstract

Wild animals represent a constant source of *Trichinella* spp. infections for domestic animals and humans. To date, four species of *Trichinella* have been isolated in wild boar populations in Europe: *T. pseudospiralis*, *T. spiralis*, *T. britovi* and *T. nativa*, in addition to several mixed infection types and one hybrid formation between *T. britovi* and *T. spiralis*. Meanwhile, insufficiently thermally processed wild boar meat has been reported to be a source of trichinellosis in humans in several European countries. In Croatia, there have been no reported or proven cases of trichinellosis caused by wild boar meat consumption. The aim of this study was to obtain data on the prevalence of *Trichinella* species present in Croatia and to anticipated the potential risk of infection for humans in specific Croatian regions based on information obtained over an eight-year surveillance period. A veterinary inspection of wild boar carcasses for *Trichinella* larvae in Croatia has been mandatory since 1989, and the artificial digestion method was introduced as a compulsory test for wild boar samples in 2008. Based on the official data submitted to the Ministry of Agriculture, Directorate of Veterinary Services, in the period 2010–2017, 303 of 183,184 (0.17%) wild boar meat samples tested positive for *Trichinella* spp. Infected wild boar were found in 18 of 21 counties. Of these positive samples, 85 were submitted by the authorised veterinary inspectors to the National Reference Laboratory for further examination. The intensity of infection in muscle samples was 0.04–152.66 (mean: 23,37) larvae per gram, and *Trichinella* species were identified as *T. spiralis*, *T. britovi*, *T. pseudospiralis* and *T. spiralis* + *T. britovi*. Genetic analysis of *T. pseudospiralis* isolates demonstrated their belonging to the Palaearctic population.

## Highlights

• In the 2010-2017 period, 303 out of a total of 183,184 (0.17%) wild boar samples were found positive for *Trichinella* spp.
• Involved *Trichinella* species were identified as *T. spiralis, T. britovi, T. pseudospiralis* and *T. spiralis+T. britovi.*
• Genetic analyses of *T. pseudospiralis* isolates confirm their belonging to the Palaearctic population.
• In Croatia, there have been no reported or proven cases of trichinellosis caused by wild boar meat consumption.
• Inspection for *Trichinella* of shot wild boar carcasses in Croatia is mandatory since 1989.

## 1 Introduction

The life cycle of parasites from the genus *Trichinella* occurs in two separate cycles: the domestic and sylvatic cycles. Although both cycles function independently of each other, incidental crossovers have been reported in many cases worldwide. The explanations for this phenomenon is attributed to the role of synanthropic animals and human behaviour [1,2].

Wild boar is a wildlife species in which infections have been reported very often in different parts of Europe and the world. The omnivorous diet of the wild boar and its wide geographical distribution in Eurasia can explain this. In Europe, four species of *Trichinella* are known, with numerous cases of mixed infections (International *Trichinella* Reference Centre, ITRC), indicating frequent contacts between boars and sources of *Trichinella* infections. Wild boar may also serve as a reservoir of infection for other carnivorous or omnivorous wild animals and birds, thereby enabling the spread of *Trichinella* into distant parts of the world [3]. The wild boar population in Croatia is an indicator of the presence and distribution of *Trichinella* spp. parasites, for several reasons: wild boars are present throughout Croatia, wild boar hunting is a widespread activity in all counties, and the inspection of wild boar samples for *Trichinella* prior to consumption has been compulsory since 1989 [4]. Furthermore, wild boar meat is believed to be the second most significant source of infection in humans by parasites of the genus *Trichinella* [1]. In Croatia to date, *Trichinella* infection has been recorded in pigs and wolves. Despite the limited territory of Croatia, short time frames and small sample sizes, a link between the domestic and sylvatic *Trichinella* cycles was found in two studies [5,6].

In the 1990s, trichinellosis in humans became a significant public health issue in Croatia. Epidemics, in addition to sporadic cases, occurred as a consequence of consumption of insufficiently thermally processed pork products in the winter months following the pig butchering season, without prior testing for *Trichinella* infection [7]. Though there are no records of human infections in Croatia caused by the consumption of wild boar meat to date, the possibility of infection exists, and therefore the objective of this study was to determine the specificities of *Trichinella* infection in wild boar populations, and to use this information to identify areas at risk and potential pathways of infection.

The discovery of two isolates of *T. pseudospiralis* species in the wild boar population has prompted us to more deeply investigate the occurrence of this rare species in our country. We analysed the sequence of the expansion segment V (ESV) region of the genomic lsrDNA gene to establish the origin of isolates and their relationship with isolates from other zoogeographic regions.

## 2 Materials and methods

Data of tested and *Trichinella*-infected wild boar samples in an eight-year period (2010–2017) were submitted to the Ministry of Agriculture by licensed veterinary organisations. These data were key for calculating the number of tested samples, the number of positive counties and the ratio of positive to overall tested samples.

Data on the number of hunted wild boar per year were taken from the statistical yearbook of the Central Bureau of Statistics (2010–2017) [8]. The data about the number and the origin of the infection with different *Trichinella* spp. in wild boars and other animals for some EU countries were taken from ICTR [9].

Samples from wild boar submitted for testing to authorised veterinary laboratories originated from the diaphragm pillar or, in its absence, the intercostal or foreleg muscles. All samples were tested using the artificial digestion method pursuant to EU Regulation No 2015/1375. Of the positive samples, 85 were submitted to the National Reference Laboratory for further testing, where artificial digestion was repeated and infection level determined. Isolated larvae were fixed in 96% alcohol and stored at 2°–8 °C (36°–46 °F) until PCR was conducted. The identification of *Trichinella* species was performed with the multiplex PCR [10,11] method at the European Reference Laboratory for Parasites (EURLP; Rome, Italy) or at the National Reference Laboratory for parasites (genus *Trichinella*) of Croatia.

Two *T. pseudospiralis* isolates were sent to the EURLP for further molecular testing. The ESV region was amplified by primers: oTsr1 (5′-CGAAAACATACGACAACTGC) and oTsr4 (5′-GTTCCATGTGAACAGCAGT) [10,12]. The obtained products were sequenced and sequences were aligned using the Clustal W program from DS gene.

The number of tested and positive samples were compared statistically between consecutive years and among all the years by the chi-square test.

## 3 Results

Of the total 183,184 wild boar samples, 303 tested positive for *Trichinella* larvae. An average of 0.17% of wild boars tested positive for *Trichinella* infection during the study period (annual percentage ranged from 0.09 to 0.32%) (Table 1). Statistical analysis of the data showed significant differences in the appearance of infected carcasses of wild boar between years. In comparing the appearance of *Trichinella* infections in one year with the previous year, a statistically significant difference was found between 2010 and 2011 (*p* = 0.03), between 2014 and 2013, and between 2015 and 2014.

**Table 1:** Overview of hunted wild boars and tested wild boar samples for infection with *Trichinella* spp. (2010–2017).

Year	Number of hunted wild boars	Number of tested samples	Number of positive samples	Percentage of positive samples	95% CI	Number of positive counties
2010	18,409	17,232	55	0.32	0.2350–0.4034	14
2011	21,871	21,459	45	0.21	0.1485–0.2709	14
2012	24,496	24,615	35	0.14	0.0951–0.1893	14
2013	21,436	16,297	22	0.13	0.0786–0.1914	9
2014	26,394	21,141	66	0.31	0.2370–0.3874	15
2015	26,997	27,280	25	0.09	0.0557–0.1275	10
2016	29,563	25,523	27	0.11	0.0659–0.1457	9
2017	30,000	29,637	28	0.09	0.0595–0.1295	10
Total	199,166	183,184	303	0.1654	0.1468–0.1840	

**Fig. 1:**
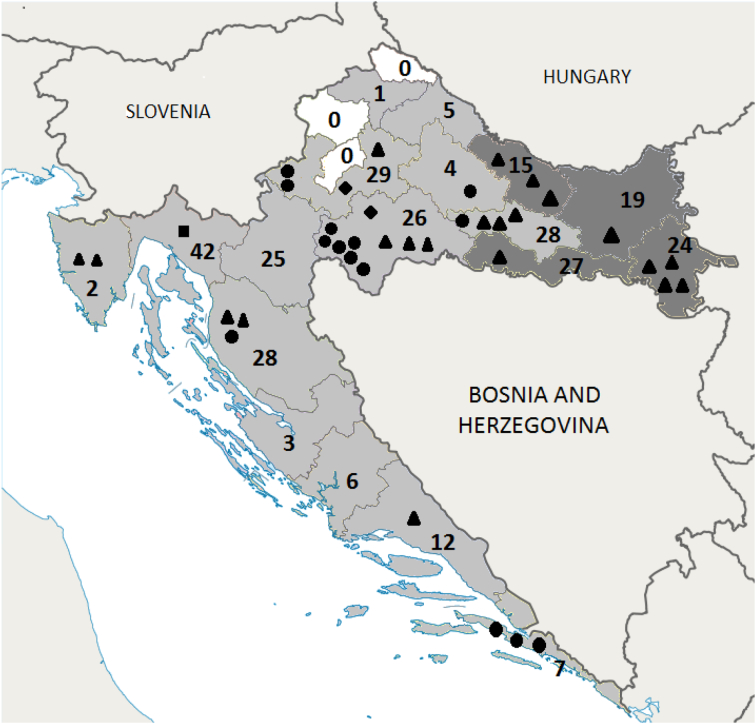
Species and origin of identified *Trichinella* spp. Croatian counties: dark grey - trichinellosis endemic counties; light grey - counties with reports of *Trichinella* infection in wild boar (2010–2017); white - counties with no reports of *Trichinella* infection in wild boar (2010–2017). Numbers indicated the number of wild boar samples positive for *Trichinella* infection within the county (2010–2017). Symbols: triangle - *T. spiralis*; circle - *T. britovi*; diamond - *T. pseudospiralis*; square - *T. spiralis* + *T. britovi*.

According to the annual reports of the Central Bureau of Statistics, a total of 199,166 wild boars were hunted in the period 2010–2017. Of the 303 positive samples, 85 were submitted to the national reference laboratory for further testing, and parasite infection with the genus *Trichinella* was confirmed in 42 of these samples (49%), with an infection level ranging from 0.04 to 152.66 larvae per gram of sample. Among the 42 confirmed *Trichinella* infection reports, molecular testing confirmed *Trichinella* spp. in 38 of these samples, which four samples did not give a successful result in the amplification of parasite genetic material. Molecular analyses indicated the presence of three *Trichinella* species: *T. spiralis* (21 samples), *T. britovi* (14 samples), *T. pseudospiralis* (2 samples) and one case of mixed infection (*T. spiralis* + *T. britovi*). Given the mean calculated mean intensity (sum of all invasion levels for each *Trichinella* spp. divided by the number of isolates of that same species), the following results were obtained: 1. *T. spiralis*: 36.82 L/g sample (range 0.4 to 152.66); 2. *T. pseudospiralis*: 20.73 L/g sample (range 8.5 to 32.96), and 3. *T. britovi*: 13.4 L/g sample (range 0.06 to 53.33). One isolate confirmed coinfection with *T. spiralis* + *T. britovi* with an infection level of 0.2 L/g sample. Fig. 1 shows the number of positive samples and the *Trichinella* species by county.

The ESV sequences of the two *T. pseudospiralis* isolates were identical to each other and identical to isolates originating from other European countries (i.e. Denmark, Finland, France, Italy and Sweden). This demonstrates that the Croatian isolates belong to the Palaearctic *T. pseudospiralis* population. Moreover, the obtained results confirm the previously reported high uniformity at the ESV locus among *T. pseudospiralis* isolates belonging to the Palaearctic population [13] and the differences at genetic level compared to isolates belonging to different zoogeographic regions (Nearctic and Australian populations).

## 4 Discussion

Wild boar is present throughout Croatia, and wild boar hunting is both a traditional activity and a recent tourism product. Due to the nutritional and caloric properties, and its culinary attractiveness, wild boar meat has become a delicacy throughout most of Europe and the world. Meanwhile, wild boar meat presents a high risk for domestic animals and humans, as it is a reservoir for numerous viruses, bacteria and parasites that can be transmitted to humans [14]. Due to the health risks of consuming wild boar meat and meat products, veterinary inspections are necessary to exclude possible infections. One of the basic tests includes an examination of a muscle tissue sample for *Trichinella* spp. infection. This testing became compulsory in Croatia in 1989, when either the compression method or the artificial digestion method were permitted [3], though since 2008 only the artificial digestion method is applied [15].

In this study, *Trichinella* infection in wild boar was confirmed in 18 of 21 counties. *T. spiralis* was the most widely distributed species of *Trichinella* spp. in Croatia, found in 11 counties, while *T. britovi* was found in 7 counties. *T. pseudospiralis* was confirmed in two counties in central Croatia. In terms of the geographic distribution of *Trichinella* species, it is evident that *T. spiralis* dominated in the four easternmost counties of Croatia (the endemic region), while *T. spiralis* and *T. britovi* were equally represented elsewhere in the country. Important epidemiological information was seen in two counties in central Croatia (Sisak-Moslavina and Zagreb County), where a relatively large number of positive wild boars were found (26 positive/34,264 tested; 29 positive/11,284 tested, respectively), with the presence of all three *Trichinella* species. It can be assumed that the situation is likely the same in the neighbouring western Karlovac County (25 positive/15,158 tested); however, no positive samples were obtained from this county for identification. For all three of these counties, they are characterised with excellent forest coverage and rivers and streams prone to occasional flooding (8).

The mean prevalence of 0.17% positive findings shows that *Trichinella* is well established in the wild boar population of Croatia, though this prevalence differed in comparison with data from Latvia (2.5%) [16], Poland (2%) [17], Germany (0.005%) [18] and Hungary (0.015%) [19]. However, since *Trichinella* infections were confirmed in only 49% of the samples submitted to the NRL, the reality is that the infection of wild boar with *Trichinella* spp. is likely lower than the calculated rates. The explanations as to why infections were not confirmed at the NRL were that nematodes not belonging to a *Trichinella* genus were found in the primary tests (as confirmed by further PCR identification) or amplifications of the nematodes failed due to improper handling of suspect samples (e. g. freezing).

A comparison of the data from the Statistical Yearbook of the Central Bureau of Statistics on the number of hunted wild boar (for 2010–2017) and the data of the Ministry of Agriculture on the number of tested samples of wild boar in the same period shows a difference of 15,982 hunted but not tested samples. This difference suggests a significant potential threat of trichinellosis if hunted wild boar are consumed without first being tested. The declining trend of positive samples of wild boar and reduced number of counties in which positive samples were recorded in a four-year period (2010−2013) ended in 2014, and already in 2015 these data were back at pre-2014 levels (Table 1). A specific occurrence in 2014 which may have directly or indirectly affected these results was the heavy rainy period in spring 2014 that resulted in severe, long-term flooding in Croatia and the surrounding countries. The assumption is that the water torrents brought illegally or inadequately deposited organic waste (e.g. carcases or hunting waste) to the surface, thereby enabling large quantities of potential sources of infection for wild boars and other wild animals.

Molecular analysis of the isolated larvae showed that the dominant *Trichinella* species in the wild boar population of Croatia, both in abundance and in geographical spread, is *T. spiralis*. Though *T. britovi* is the etiological agent of the sylvatic *Trichinella* cycle in the Palaearctic belt (including most of Europe) [1], it would appear that the results of the wild boar population do not always comply with this conclusion. The present results and reports from other European countries support this, i.e. Poland [17,20], Spain [21] and Germany [22,23]. The reasons for the prevalence of *T. spiralis* among wild boar in Croatia likely goes back to the 1990s, during the Croatian Homeland War (1991–1995) and post-war era, when *Trichinella* infected carcasses of domestic animals remained undisposed of and were a source of *Trichinella* infection in wild boar, either directly or through synanthropic animals. This most likely caused the spillover of *T. spiralis* from the domestic into the sylvatic cycle, where it has remained. The isolated finds of samples of wild boar infected with *T. spiralis* in the westernmost county and in some coastal and mountainous counties can be considered a result of the import of wild boar from endemic areas. *T. britovi* is the second most common species of the genus *Trichinella*, found in seven counties lying exclusively outside the endemic area. Though co-infection in wild game has been recorded in nine other European countries (ITRC), our finding is the first record of coinfection in Croatia. This coinfection was by *T. spiralis* + *T. britovi*, and the sample was found in the mountainous part of Croatia. While the host-pathogen interaction of wild boar infection has not been sufficiently explored [18], according to the data of ITRC, wild boar is the most frequent animal in which co-infection was recorded. Of the 65 isolates, 60 contained *T. spiralis* in combination with *T. britovi*, three isolates in combination with *T. pseudospiralis*, and two isolates found a combination of *T. nativa* and *T. britovi*. The current distribution of *T. britovi* and *T. spiralis* in wild game (wolf and wild boar) in Croatia suggests that in continental Croatia and particularly its easternmost region, *T. spiralis* is dominant in wild boar in comparison to *T. britovi*. The cause for this may be the biological “strength” of *T. spiralis*, which may inhibit infections with *T. britovi*, as shown in experimental co-infections with *T. nativa* [10] and *T. pseudospiralis* [24]. These authors confirmed that co-infections did not occur when the animal was primarily infected with *T. spiralis*. The high number of infections with *T. spiralis* in continental Croatia is also due to more frequent contact of wild boar with the domestic *Trichinella* cycle, which in recent decades has spread throughout the four easternmost counties where *Trichinella* is deemed an endemic disease [5]. The third possible proof of the biological “strength” of *T. spiralis* can be found in the average invasion level in samples infected with *T. spiralis* in comparison with those samples infected by *T. britovi,* which in this study was almost 3:1 higher for *T. spiralis*.

The discovery of the presence of *T. pseudospiralis* in the wild animal population of Croatia is a new finding, since previously this species of *Trichinella* was found in a sample originating only in a pig [25]. The route of infection for described case of the positive pig was not determined, and the authors assumed that the infection was result of the feeding on scraps. Both wild boar samples infected with *T. pseudospiralis* were found in central Croatia in 2017. These findings occurred 11 years after and about 150 km from the site of the first isolate of *T. pseudospiralis* in Croatia, and there are no evident links between the two positive samples from 2017 and the positive sample from 2006.

However, a possible source of infection for such sporadic and isolated cases could be birds. *T. pseudospiralis* has been reported in 8 avian species, and the cosmopolitan nature of *T. pseudospiralis* is another supporting factor for the influence of birds in the spread of this species to different parts of the world [26]. In a review paper from 2016, Pozio stated that reports on the presence of *T. pseudospiralis* in Europe were increasing, suggesting that there are three epidemiological events that may have contributed to the spread of this *Trichinella* species in Europe. In Croatia, a portion of the seagull population often winters in landfill areas of large cities in central Croatia, and they could be one reason for the find of this *Trichinella* species in the wild boar population. A second possible source of infection is the very numerous carnivorous and omnivorous bird population that occasionally or permanently inhabits the Lonjsko Polje Nature Park, an Important Bird Area according to the EU Birds Directive [27], and is also in the direct vicinity of the find site for the two isolates of *T. pseudospiralis*.

The classification of the Croatian *T. pseudospiralis* isolates as belonging to the Palaearctic population suggest that these isolates do not originate from distant zoogeographic areas (Nearctic or Australian regions) but that the sources of infection must be sought within the European wild fauna. The lack of information regarding animal species involved in the natural cycle of *T. pseudospiralis* can be explained by the greater effort made to investigate animals considered to be the main source of infection for humans (i.e. domestic swine and wild boar) than on efforts to examine birds and micromammals, which are also more difficult to study [26].

However, in recent periods, other possibilities for the spread of *T. pseudospiralis* around the world other than birds have arisen. In Argentina, the first case of *T. pseudospiralis* was described in domestic swine [28] and the authors listed the possibilities of the introduction of this species as the migration of birds, or import of wild and/or domestic swine at the time of European colonisation. In the United Kingdom, Learmont [29] describe an isolated case of *T. pseudospiralis* in fox, which was found to be positive among numerous negative samples in a testing campaign that continued until 1999. In the case, infection could be attributed to the feeding of foxes with remnants of meat products imported from abroad, or via infected rats that may have arrived via ship to nearby ports and later become prey to the foxes.

There is still no definitive explanation for the “sudden” and isolated discoveries of *T. pseudospiralis* in different parts of the world.

## 5 Conclusions

*Trichinella* infection in wild boar in Croatia reveals the pretty steady prevalence of positive samples throughout the observed period. The discoveries of the presence of non-capsulated species of *Trichinella* and the first co-infection in the wild boar population indicate similar findings in other European countries. The opportunity for trichinellosis caused by consumption of wild boar meat can be considered present in all parts of the country, and particular attention should be focused on counties in central and eastern Croatia. Since there are some still unknown features of the circulation of *T. pseudospiralis* in nature and it is proven that the bird population can be infected with *T. pseudospiralis*, it pushes us to investigate in this direction.

## Declaration of Competing Interest

The authors have no conflicts of interest to declare.
